# Arterial spin labeling CMR perfusion imaging is capable of continuously monitoring myocardial blood flow during stress

**DOI:** 10.1186/1532-429X-17-S1-P145

**Published:** 2015-02-03

**Authors:** Hung P Do, Ahsan Javed, Terrence R Jao, Hee-won Kim, Andrew J Yoon, Krishna S Nayak

**Affiliations:** Department of Physics and Astronomy, University of Southern California, Los Angeles, CA USA; Ming Hsieh Electrical Engineering Department, University of Southern California, Los Angeles, CA USA; Department of Biomedical Engineering, University of Southern California, Los Angeles, CA USA; Department of Radiology, University of Southern California, Los Angeles, CA USA; Department of Medicine, University of Southern California, Los Angeles, CA USA

## Background

Myocardial arterial spin labeling (ASL) is a non-invasive non-contrast CMR perfusion imaging technique [Zun et al., '09] that is compatible with adenosine stress testing [Zun et al., '11]. Here, we demonstrate its ability to continuously monitor MBF during stress, which has a variety of potential applications including interventional CMR and the study of endothelial function. We used mild sustained isometric handgrip stress.

## Methods

### Imaging methods

Three healthy subjects (25-30, 3 males, non-smoker, no family history of CAD) were scanned on a GE 3T scanner. A previously described myocardial ASL sequence [Zun et al., '09; Zun et al., '11; Do et al., '14] was run continuously for roughly 20 minutes, in a free-breathing mode with respiratory triggering, on a single mid short-axis slice. The imaging protocol was divided into three stages: 6 min rest, 6 min stress, and 6-8min recovery. Imaging parameters were TE/TR=1.5/3.2ms, FOV = 240-320mm, matrix size = 96x96, slice thickness = 10mm, and an acceleration factor of 2. Stress was induced using a sustained isometric handgrip exercise at 30% of each subject's maximum voluntary contraction [Brenkenhoff et al., '13].

### Data analysis

Motion correction [Avants et al., '08] was used to generate myocardial masks of all acquired control and tagged image pairs from a manually segmented reference pair [Javed et al., '15 (submitted)]. A sliding-window of 12 image pairs, corresponding to 4-5 minutes, were used to generate the MBF value at each time point.

## Results

**Figure 1:**
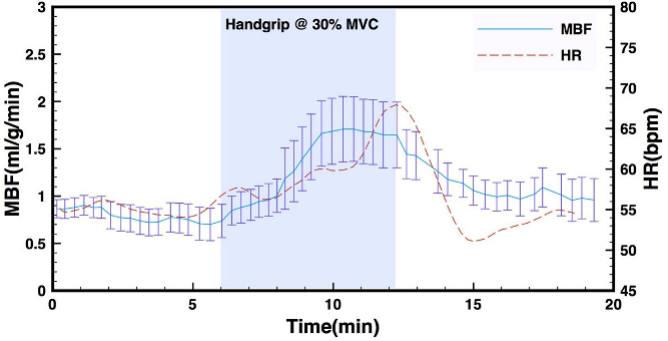
**MBF (blue) and HR (red) as a function of time for one experimental subject.** The shaded area represents duration of handgrip stress at 30% of maximum voluntary contraction.

Figure 1 shows MBF and heart rate (HR) response from the experiment with the lowest physiological noise, and the most stable heart rate during the initial rest period. MBF and HR increased after the onset of stress and also recovered to approximately the same values as during the initial rest period.

## Conclusions

This study demonstrates the feasibility of noninvasive and continuous monitoring of MBF using myocardial ASL with mild stress. Because MBF is related to certain factors such as HR, stroke volume, oxygen demand and vessel dilation, this ASL approach could be used for continuous monitoring of both perfusion and endothelial function.

## Funding

American Heart Association; Wallace H. Coulter Foundation.

